# *Wernerius inyoensis*, an elusive new scorpion from the Inyo Mountains of California (Scorpiones, Vaejovidae)

**DOI:** 10.3897/zookeys.177.2562

**Published:** 2012-03-23

**Authors:** Michael M. Webber, Matthew R. Graham, Jef R. Jaeger

**Affiliations:** 1 School of Life Sciences, University of Nevada Las Vegas, 4505 South Maryland Parkway, Las Vegas, Nevada 89154-4004, USA

**Keywords:** *Vaejovis*, taxonomy, 16S, COI, barcoding, Death Valley

## Abstract

A new scorpion species is described from the Inyo Mountains of California (USA). The presence of a strong subaculear spine, along with other characters, places the new species within *Wernerius*, an incredibly rare genus that until now consisted of only two species. *Wernerius inyoensis* **sp. n.** can be most easily distinguished from the other members of the genus by smaller adult size, femur and pedipalp dimensions, and differences in hemispermatophore morphology. Previous studies have suggested that the elusive nature of this genus may be attributed to low densities and sporadic surface activity. Herein, we provide another hypothesis, that *Wernerius* are primarily subterranean. Mitochondrial sequence data are provided for the holotype.

## Introduction

The discovery that scorpions fluoresce under ultra-violet light (Stahnke 1972) marked a revolution in our knowledge of scorpion diversity. Before then, only the more commonly encountered species, such as those that are large in size or especially abundant, were well-known. Subsequent to this discovery, scorpions began to accumulate in biodiversity collections, and a flurry of new scorpion taxa were described worldwide. Numerous new scorpion species are still being discovered in places as seemingly well-studied as the United States (Graham 2007, Ayrey 2009, Ayrey 2011, Ayrey and Soleglad 2011, Sissom 2011, Soleglad et al. 2011) and Mexico (i.e. Contreras-Félix and Santibáñez-López 2011; Francke and Ponce-Saavedra 2010; Graham and Bryson 2010; Santibáñez-López and Francke 2010; Santibáñez-López and Sissom 2010).

Herein, we describe another new species of scorpion from southwestern North America that may never have been discovered without the use of ultra-violet light. The new species is represented by a single male collected from the Inyo Mountains of eastern California in 2009. Like the majority of recently discovered scorpions in North America, this species is particularly small (~16 mm) and was almost overlooked during our survey. The new species possesses a strong subaculear spine and other characters representative of *Wernerius* (Soleglad and Fet 2008), a notoriously elusive genus. Until now, the genus was comprised of only two species, *Wernerius spicatus* (Haradon 1974) from the southwestern portion of Joshua Tree National Park, and *Wernerius mumai* (Sissom 1993) from rock-strewn habitats along the Colorado River near Parker, Arizona. Additional efforts to recollect these species (Sissom 1993), and our attempt to collect additional samples of the new species from the type locality proved futile.

**Figure 1.**
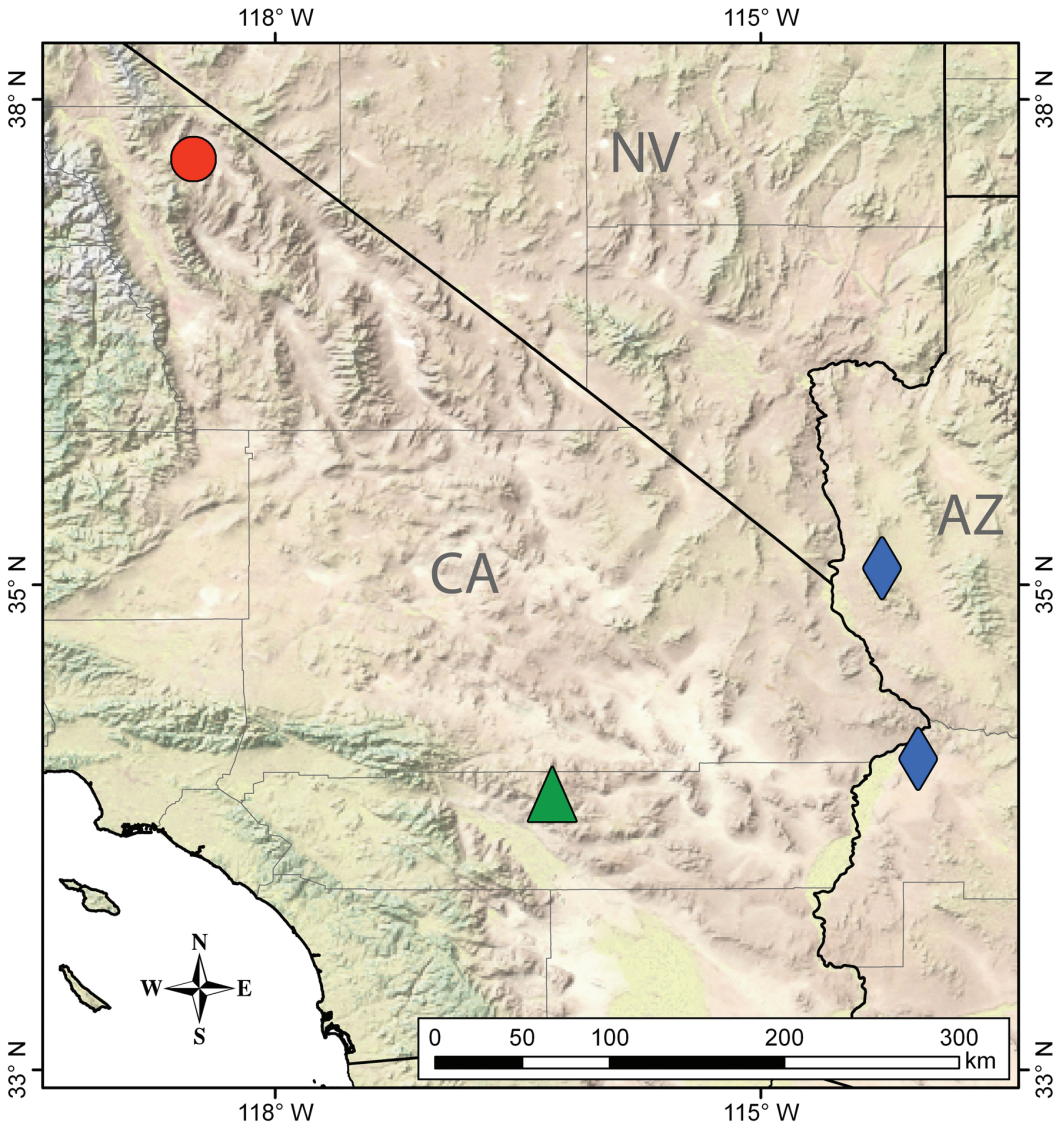
Distribution of *Wernerius inyoensis* sp. n. (red circle, Inyo Mountains, California) and closely related species: *Wernerius spicatus* (green triangle, San Bernardino Mountains, California) and *Wernerius mumai* (blue diamond, Parker and Black Mountain, Arizona).

The discovery of *Wernerius* in the Inyo Mountains extends the range of the genus over 400 kilometers to the north, making its known distribution extremely disjunct (Fig. 1). We suspect that the three species of *Wernerius* must either occur at low densities, exhibit sporadic surface activity, or live subterraneously (or a combination of these) making these scorpions some of the most enigmatic and little-known in North America. For these reasons, and even though based on only a single specimen, the description of this new species is an important contribution to the growing knowledge of vaejovid scorpions. Since all three species of *Wernerius* are incredibly rare, we also sequenced portions of two mitochondrial genes to support DNA barcoding initiatives (Ratnasingham and Hebert 2007) and to assist with ongoing work on the systematics of family Vaejovidae.

## Material and methods

Measurements are as described by Stahnke (1970), trichobothrial patterns are as in Vachon (1974) and Soleglad and Fet (2003), and pedipalp finger dentition follows Soleglad and Sissom (2001). Total lengths were measured from the anterior margin of the carapace to the aculeus tip with the telson fully extended.

Acronyms of depositories.— DEVA, Death Valley National Park, USA; SDNHM, San Diego Natural History Museum, San Diego, California, USA.

### Molecular analysis

Total genomic DNA was extracted from leg and pedipalp tissue from the left side of the holotype using a DNeasy Extraction Kit (Qiagen Inc.), leaving the right side of the voucher intact. A portion of the cytochrome oxidase subunit I (COI) gene was amplified with forward primer COI_modF (5’- ATCATAAGGATATTGGGACTATGT - 3’, Bryson et al. in review) and reverse primer C1_2776_MOD (5’- GGATAATCAGAATAHCGAGG -3’). A section of the 16S ribosomal RNA gene was amplified using scorpion-specific primers (Gantenbein et al. 1999). Double-stranded cycle sequencing was performed using the same primers and the Big Dye Terminator v. 3.1 Cycle 6 Sequencing Kit (Applied Biosystems). The COI sequence was submitted to the Barcode of Life Data system (Ratnasingham and Hebert 2007), and both COI and 16S data were submitted to GenBank (Accession numbers JQ727686 and JQ727686).

## Taxonomy

### Family Vaejovidae Thorell, 1876

**Subfamily Syntropinae Kraepelin, 1905**

**Tribe Stahnkeini Soleglad & Fet, 2006**

**Genus *Wernerius* Soleglad & Fet, 2008**

#### Wernerius inyoensis sp. n.

urn:lsid:zoobank.org:act:A50D0CA5-CBE7-4C5F-9BC6-EFB155F7EFA2

http://species-id.net/wiki/Wernerius_inyoensis

Figs 1 [Fig F2] [Fig F3] [Fig F4] 14

##### Type material.

United States: *California*: male holotype, Loretta Mine Road, Inyo Mountains, Death Valley National Park, 37.2299°N, 117.9568°W, 1706 m, 9 September 2009, M.R. Graham and G.M. Graham Jr. (DEVA 54174).

##### Etymology.

The specific epithet refers to the type locality in the Inyo Mountains, California.

##### Diagnosis.

Small in size, with the only known adult male less than 17 mm in total length. Yellow-orange base color with darker carinae on the pedipalps, and segments of the metasoma. Genital operculum divided below posterior one fifth, carapace very slightly emarginate; pectine count 11–11; 7 inner (ID) denticles on the pedipalp movable finger and 6 on the fixed finger.

Although specimens of *Wernerius spicatus* and *Wernerius mumai* were not available for study (Sissom pers. comm.), based on the original descriptions of these species (Haradon 1974, Sissom 1993), it appears that *Wernerius inyoensis* sp. n. is morphologically most similar to *Wernerius spicatus*. Both species share ID denticle counts, have similar femur and patella L/W ratios, and overlap in pectine tooth counts. However, *Wernerius inyoensis* sp. n. differs from *Wernerius spicatus* by having larger metasomal and pedipalp dimensions. *Wernerius inyoensis* sp. n. also differs from *Wernerius spicatus* in hemispermatophore morphology. The length of the lamellar hook in *Wernerius inyoensis* sp. n. is relatively short and the dorsal trough is shallow as indicated by the ratio of the lamellar hook length/length of the entire lamina (0.338) when compared to that of *Wernerius spicatus* (0.439). In addition, the basal constriction is less well-defined in *Wernerius inyoensis* sp. n. as indicated by the ratio of width at lamellar hook/lamina length (*Wernerius inyoensis* sp. n. = 0.169; *Wernerius spicatus* = 0.123). The distal end of the lamina is also straighter and wider than *Wernerius spicatus*, which exhibits a slight curve and tapers posteriorly (ratio of width at distal end of lamina/width at lamina midpoint; *Wernerius inyoensis* = 0.900, *Wernerius spicatus* = 0.652). The ratio of the total width of the lamina at the midpoint/inner surface of the lamina groove to the right lateral surface of the lamina, indicates that the lamellar spine of *Wernerius inyoensis* sp. n. (1.33) is wider than that of *Wernerius spicatus* (1.09).

**Figure 2. F2:**
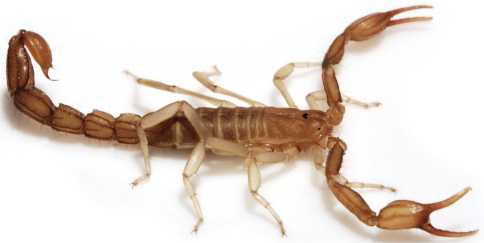
*Wernerius inyoensis* sp. n. *in vivo*.

*Wernerius inyoensis* sp. n.can be distinguished from *Wernerius mumai* by the following combination of characters: smaller adult size (of the holotype, < 17 mm), more robust femur (L/W ratio 3.54) and a shorter, thinner pedipalp, 5 OD denticles on the pedipalp movable finger in addition to 5 on the fixed finger, and ventral metasomal setae counts. Inframedian carinae are crenulate and complete on metasomal segment I, and cover the posterior half of metasomal segments II and III. A comparison of characters is provided in Table 1.

**Table 1.** Measurements (in millimeters) of all known adult specimens in genus *Wernerius*.

	***Wernerius inyoensis* sp. n.**	***Wernerius spicatus***	***Wernerius spicatus***	***Wernerius spicatus***	***Wernerius mumai***
Sex	Male	Male	Female (Holo)	Female (Para)	Female (Holo)
Total Length	16.4	15.9	17.3	16.1	24.5
Carapace Length	2.38	2.2	2.35	2.25	3.5
Mesosoma Length	4.89	4.95	6.1	5.65	8.5
Metasoma Length	7.48	6.65	6.6	6.05	9.15
Met I Length	1.05	0.95	0.9	0.8	1.35
Met I Width	1.29	1.1	1.15	1.1	1.8
Met I L/W	0.81	0.86	0.78	0.73	0.75
Met I Diameter	1.12	N/A	N/A	N/A	N/A
Met II Length	1.19	1.05	1.05	0.95	1.5
Met II Width	1.31	1.1	1.15	1.1	1.8
Met II Diameter	1.10	N/A	N/A	N/A	N/A
Met III Length	1.24	1.1	1.15	1.05	1.6
Met III Width	1.40	1.15	1.15	1.1	1.85
Met III L/W	0.89	0.96	1.0	0.95	0.86
Met III Diameter	1.05	N/A	N/A	N/A	N/A
Met IV Length	1.71	1.5	1.35	1.25	2.2
Met IV Width	1.50	N/A	1.25	1.2	2.1
Met IV Diameter	1.07	N/A	N/A	N/A	N/A
Met V Length	2.29	2.05	2.15	2	2.6
Met V Width	1.45	1.4	1.3	1.2	2.05
Met V L/W	1.58	1.46	1.65	1.67	1.27
Met V Diameter	1.07	N/A	N/A	N/A	N/A
Telson Length	2.27	2.1	2.25	2.15	3.35
Vesicle Length	1.67	1.5	1.65	1.6	2.55
Vesicle Width	1.10	1.05	1.05	1.15	1.85
Vesicle L/W	1.52	1.43	1.57	1.39	1.38
Vesicle Diameter	0.76	0.75	0.8	0.8	1.3
Aculeus Length	0.60	0.55	0.6	0.55	0.8
Pedipalp Length	7.95	6.95	N/A	N/A	11.6
Femur Length	2.02	1.95	2	1.9	3
Femur Width	0.57	0.55	0.6	0.55	0.95
Femur L/W	3.54	3.55	3.33	3.45	3.16
Patella Length	2.36	2.15	2.3	2.15	3.25
Patella Width	0.64	0.6	0.7	0.65	1.1
Patella L/W	3.69	3.58	3.29	3.31	2.95
Chela Length	3.57	2.85	3.65	3.5	5.35
Chela L/W	3.84	3.35	3.65	3.50	3.45
Palm Length	1.98	N/A	1.85	1.8	N/A
Palm Width	0.93	0.85	1	1	1.55
Palm Diameter	1.12	0.95	N/A	N/A	1.65
Movable Finger L	2.24	2.05	2.2	2.1	3.15
Fixed Finger L	1.79	1.6	1.8	1.7	2.45
Pectine count	11,11	12,12	11,11	10,11	N/A
Middle lamellae count	5,6	N/A	6,6	6,6	N/A
Fixed Finger L/Carapace L	0.75	0.73	0.77	0.76	0.70

##### Description of holotype.

*Color*: Carapace, tergites, femur, patella, and metasoma have a yellow-orange base color with dark brown to black markings on the chela and along carinae of the metasoma. Legs are yellow and slightly lighter in color than the rest of the body. Pedipalp chela is yellow-orange in color with darker reddish-brown coloration at the anterior portion of the palm where the fixed finger and movable finger meet. Chelicerae are yellow with mottling on distal half. Telson is dark-yellow to orange bordered by dark brown carinae. Pectines and genital operculum are light yellow.

##### Morphology.

Carapace: anterior margin very slightly emarginate, with three lateral eyes on each side; moderately convex dorsolaterally; finely granular with scattered small granules, with larger granules symmetrically flanking the median furrow; median furrow is slight and traverses length of carapace, excluding the median eyes; ratio of median eyes location (from anterior edge)/carapace length = 0.32; carapace length/width at median eyes = 1.44. Tergites: slightly granular with weak median carinae from distal half of tergite I, and terminating at the middle of segment VII; strong granular dorsolateral and lateral supramedian carina on posterior 4/5s of VII; pretergites very finely granular. Sternites: I–V smooth to very finely granular and without carinae; V with granular ventral lateral carinae on posterior 1/5 to posterior 3/5. Spiracles: ovoid with median side parallel to posterior sternite margin. Genital Operculum: sclerites separated on posterior 1/5 with genital papillae protruding slightly beyond posterior of operculum plates. Pectines:tooth count 11/11; middle lamellae 5/6. Metasoma: ratio of segment I length/width 0.81; segment II length/width 0.91; segment III length/width 0.89; segment IV length/width 1.14; segment V length/width 1.58. Segments I–IV: dorsolateral carinae are strong and serrate, with distal denticle of I–IV enlarged and spinoid; denticle size is largest on segments III and IV and smaller on segments I and II; possesses intermediary carinae on segments I, II, and III; inframedian carinae are crenulate, and traverse the entire length of segment I, and ½ of the posterior portion of segments II and III, lateral supramedian carinae I–III possesses serrated granules and enlarged spinoid distal denticle; carinae of segment IV are less pronounced, crenulate to serrate, and flared on distal terminus; a space exists between the dorsolateral and supramedian carinae of segments I–III, and 1/3 of segment III; intermediary carinae are less distinct and are more granular than the ventrolateral carinae; ventral carinae are weakly serrate, but less distinct than dorsal carinae; ventrolateral carinae I strong, crenulate to serrulate; on II–III serrulate to serrate; on IV crenulate to serrate; ventral submedian setae 3/3:3/3:3/3:3/3:3/3. Segment V: dorsolateral carinae moderate, granular; lateromedian carinae moderate and granular on anterior 4/5, obsolete on distal 1/5; ventrolateral and ventromedian carinae crenulate to weakly serrate; intercarinal spaces are finely granular; ventrolateral setae 2/2:2/2:2/2:2/2:3/3.

**Figures 3–10. F3:**
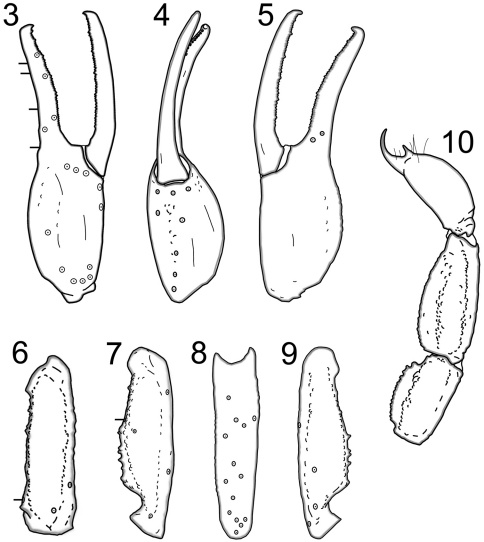
Trichobothrial patterns of *Wernerius inyoensis* sp. n. based on male holotype **3** Right pedipalp chela, external **4** Right pedipalp chela, ventral **5** Right pedipalp chela, internal **6** Right pedipalp femur, dorsal **7** Right pedipalp patella, dorsal **8** Right pedipalp patella, internal **9** Right pedipalp patella, ventral **10** Lateral aspect of metasomal segments IV and V, and telson.

**Figures 11–12. F4:**
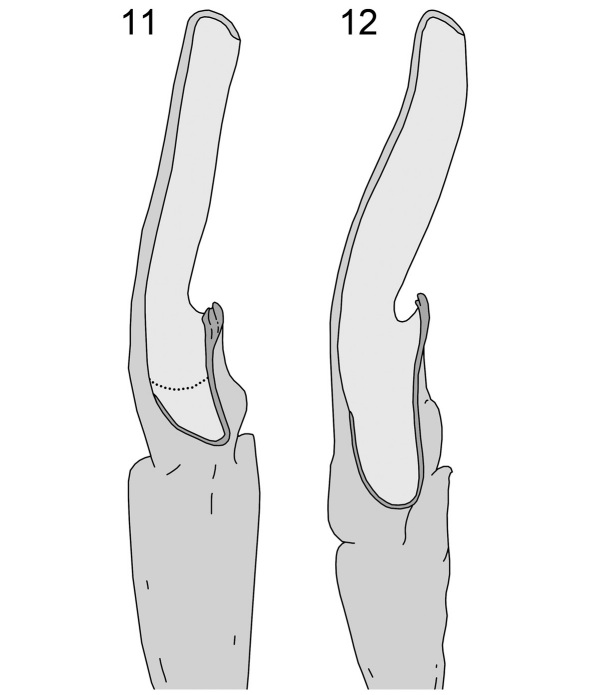
Dorsal aspect of right hemispermatophore: **11** *Wernerius inyoensis* sp. n. (dotted line indicates the ventral trough) **12** *Wernerius spicatus* (redrawn from Sissom (1993))

Telson: smooth to slightly granular with very pronounced subaculear tubercule; 3/1 LAS denticles (Fet et al. 2006). Chelicerae:dorsal edge of movable cheliceral finger with two subdistal (sd) denticles; ventral edge smooth to well developed serrula on distal 2/3. Pedipalps:trichobothrial pattern type C (Figs 3–9); ratio of chela length/width 3.84; femur length/width 3.54; patella length/width 3.69; fixed finger length/carapace length 0.36. Chela:carinae weak and smooth except for a few weak to moderate granules on D4 and D5; median (MD) denticles of fixed finger aligned and divided into six subrows by five outer (OD) denticles flanked by six inner (ID) denticles; movable finger with six subrows, five OD denticles and seven ID denticles; movable finger shorter than the carapace and slightly shorter than metasomal segment V. Femur: dorsoexternal, dorsointernal, and ventrointernal carinae denticulate, ventroexternal is slightly serrate; internal surface covered with small granules throughout. Patella:internal carinae are granulose with 5 dentate denticles; all other carinae weak to non-existent. Legs: ventral surface of tarsus with single median row of spinules terminating distally with one spinule pair. Hemispermatophore (Figs 11–12): the specimen has a wide hemispermatophore trunk with a well defined truncal flexure; the dorsal trough is shallow, with its base terminating at the distal end of the truncal flexure and tapers posteriorly; the lamellar hook is relatively large and strongly bifurcated at the distal tip, and also possesses a strong groove and slight basal constriction; the length of the lamellar hook is relatively short and the dorsal trough is shallow as indicated by the ratio of the lamellar hook length to the length of the entire lamina (0.338).

##### DNA barcode (COI)

– GCTTCTATGGTAGGGACAGCTTTGAGAT TAATAATTCGTATTGAGATTGGAAGTCCTGGGTCTTTTATTGGAGA TGATCAAATTTATAATGTTGTTGTTACTGCTCATGCTTTTGTAAT GATTTTTTTTATGGTAATACCAATTATAATTGGAGGTTTTGGAAATTG GTTAGTCCCTTTAATGTTGGGGGCTCCTGATATGGCTTTCCCTCGTT TAAATAATATAAGTTTTTGGTTATTACCTCCTGCATTTTTTTTATTATT AGGGTCAGCTTCATTGGAAAGAGGCGCAGGGACAGGCTGAACTGT GTACCCGCCTCTTTCCTCATATATGTTCCATTCTGGTGGTTCTGTT GATATGACTATTTTTTCTTTACATTTAGCTGGAGTTTCTTCAATTT TAGGAGCTATTAATTTTATTACTACTATTTTAAATATACGTATAAGTG GAATATTATTGGAGCGTATTCCTTTGTTTGTATGATCTGTAAGGAT TACTGCTATTTTATTACTTCTTTCTCTTCCCGTTCTTGCAGGGGC TATTACTATACTATTAACTGATCGAAATTTTAATACTTCTTTTTTT GATCCTGCAGGAGGGGGAGATCCCATTTTATATCAGCATT TATTTTGATTTTTTGGACATCCTGAAGTTTATATTTTAATTCTTC CTGGGTTTGGAATGGTTTCTCATATTATTAGTCATCATACTG GAAAGAGGGAGCCTTTTGGAGCTTTGGGAATGATTTATGCAATG GTTGCTATTGGGTTTTTAGGATTTGTTGTTTGGGCTCATCATAT GTTTACTGTTGGAATAGATGTTGATACTCGAGCTTATTTTACT GCTGCTACTATGGTTATTGCTGTTCCTACTGGGATCAAAATTTT TAGATGATTAGCTACTTTACATGGTTCTTATTTTGTCTTTACGC CCCCTCTTTTGTGGGCTTTGGGATTTGTTTTTCTATTTACTG TAGGAGGTTTAACTGGTGTAATTTTAGCTAATTCTTCTTTGGA TATTGTTCTTCATGATACTTATTATGTTGTAGCTCATTTTCAT TATGTTTTGTCTATAGGAGCAGTTTTTGCCATTATTGCTGGAATT GTTGAATGGTTTCCTCTATTTTTAGGTTGTCAGATGAGTGAGCG TATATTAAAAATTCATTTTTTTGTGATGCTTTTGGGGGTAAAT

##### Mensuration (mm).

Male holotype: total length 16.4; carapace length 2.38; mesosoma length 4.89; metasoma length 7.48 (excluding telson); Metasoma: segment I length/width 1.05/1.29; segment II length/width 1.19/1.31; segment III length/width 1.24/1.40; segment IV length/width 1.71/1.50; segment V length/width 2.29/1.45. Telson: length 2.27; vesicle length/width/depth 1.67/1.10/0.76; aculeus length 0.60. Pedipalps: total length 7.95; femur length/width 2.02/0.57; patella length/width 2.36/0.64; chela length 3.57; palm length/width/depth 1.98/0.93/1.12; movable finger length 2.24; fixed finger length 1.79.

##### Distribution.

Known only from the type locality in the Inyo Mountains of California where it was collected at an elevation of 1706 m.

##### Subterranean hypothesis.

The southwestern United States is one of most well-studied areas in the world in terms of scorpions, so it is puzzling that a genus as widespread as *Wernerius* is so infrequently encountered. Previous authors have attributed their rarity to low densities or sporadic surface activity (Sissom 1993), but we provide a third potential explanation, that *Wernerius* are primarily subterranean.

**Figures 13–14.**
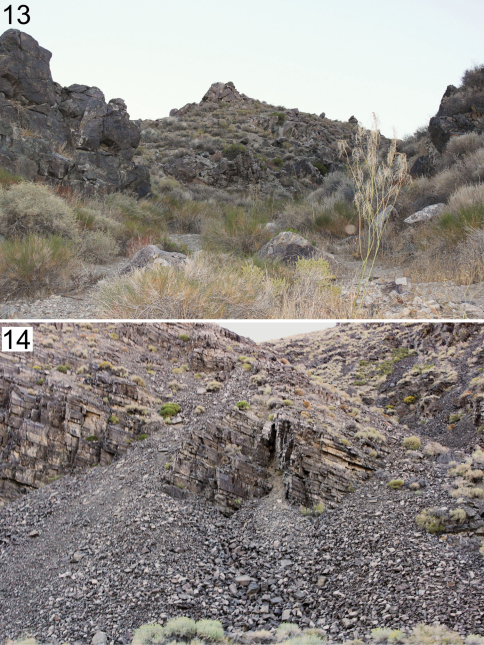
Type locality of *Wernerius inyoensis* sp. n. **13** Desert wash where the species was first discovered. **14** Talus slope that might provide a subterranean habitat for *Wernerius inyoensis* sp. n.

Recent studies of invertebrates inhabiting the deep soil strata (euedaphon) in Bulgaria have revealed a rich spider fauna (Deltshev et al. 2011). Incredibly, the different soil strata each contained a unique assemblage of spiders, many of which exhibited various degrees of morphological adaptations to underground environments. One species, *Zangherella relicta* (Kratochvíl 1935), was only found within the deepest strata surveyed. We hypothesize that *Wernerius inyoensis* sp. n. may inhabit similar environments in the North American Southwest, particularly areas of piled rock or talus slopes (Fig. 13, 14). Despite the fact that *Wernerius* are uncommonly encountered, two other lines of evidence support our hypothesis. Each species has only been collected from extremely rocky habitats, and each species is incredibly small (< 25 mm), perhaps enabling them to easily maneuver within the interstitial spaces of piled rock and talus. If true, then perhaps these small and mysterious scorpions occur at higher densities across a much wider distribution than currently known.

## Supplementary Material

XML Treatment for Wernerius inyoensis
